# Designing small molecules that target a cryptic RNA binding site via base displacement

**DOI:** 10.21203/rs.3.rs-5836924/v1

**Published:** 2025-01-29

**Authors:** Robert Batey, Lukasz Olenginski, Aleksandra Wierzba, Shawn Laursen

**Affiliations:** University of Colorado Boulder; University of Colorado Boulder; University of Colorado Boulder; University of Colorado Boulder

**Keywords:** RNA structure, drug discovery, small molecules, structure-based design, base displacement

## Abstract

Most RNA-binding small molecules have limited solubility, weak affinity, and/or lack of specificity, restricting the medicinal chemistry often required for lead compound discovery. We reasoned that conjugation of these unfavorable ligands to a suitable “host” molecule can solubilize the “guest” and deliver it site-specifically to an RNA of interest to resolve these issues. Using this framework, we designed a small molecule library that was hosted by cobalamin (Cbl) to interact with the Cbl riboswitch through a common base displacement mechanism. Combining in vitro binding, cell-based assays, chemoinformatic modeling, and structure-based design, we unmasked a cryptic binding site within the riboswitch that was exploited to discover compounds that have affinity exceeding the native ligand, antagonize riboswitch function, or bear no resemblance to Cbl. These data demonstrate how a privileged biphenyl-like scaffold effectively targets RNA by optimizing π-stacking interactions within the binding pocket.

## Introduction

The design and discovery of small molecules that selectively target RNA is a longstanding problem in chemical biology. This emerging field has the potential to develop chemical probes of RNA function and therapeutics to treat RNA-mediated disease.^1–3^ Despite ongoing efforts, there is only one FDA-approved small molecule that targets RNA outside the ribosome, risdiplam.^4^ This reinforces the notion that targeting RNA is difficult and often limited by the lack of druggable RNA targets and/or quantitative structure-activity relationship (QSAR) studies.^1–3^ Compared to proteins, which are routinely targeted with small molecule therapeutics,^5^ much less is known about the principles governing RNA-ligand interactions to guide discovery efforts.

**Figure 1:**
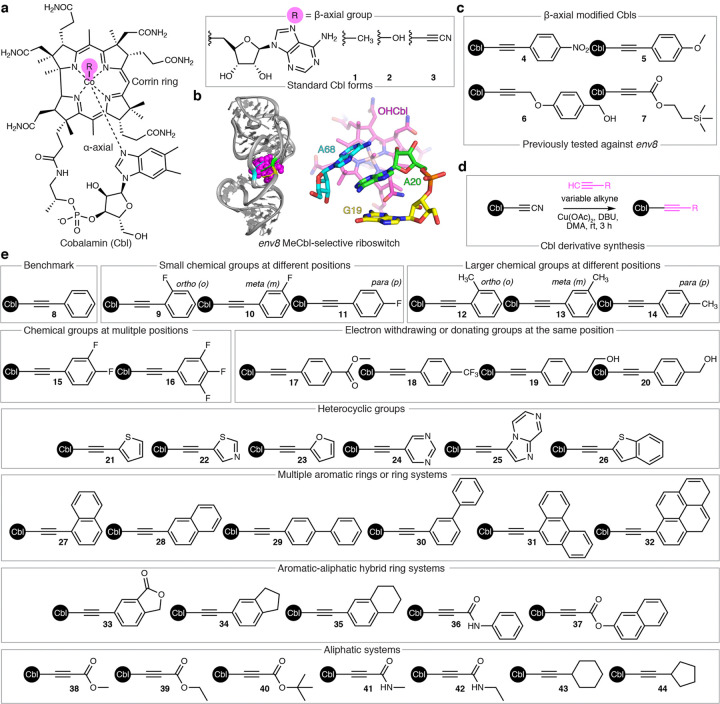
Motivation and design of our expanded Cbl derivative library. **a**, Chemical structure of standard Cbls with their corresponding β-axial groups shown. **b**, Co-crystal structure of the *env8*-OHCbI complex (PDB 4FRG^20^). Global RNA architecture (left) is displayed as cartoon, nucleotides critical for recognition of the variable β-axial group are shown in cyan (A68), green (A20), and yellow (G19), and OHCbl is represented by magenta Van der Waals spheres. The ligand binding pocket (right) consists of a π-stacking network between nucleotides G19-A20 and A20-A68 that is unimpeded by the small (-OH) β-axial group. **c**, Simplified chemical structures of previously characterized^21^ β-axial-modified Cbls **4–7** tested against *env8*. **d**, Reduction-free synthetic route^26,27^ to make Cbl derivatives from CNCbl using a variable alkyne. **e**, Overview of our expanded β-axial-modified Cbl library.

One approach to address this knowledge gap is the application of natural RNA-small molecule interactions such as those observed in riboswitches. These bacterial mRNA elements have evolved tertiary structures to selectively bind metabolites and control gene expression,^6–8^ making them ideal models to explore RNA-ligand interactions. For example, BioRelix^9,10^ and Merck^11,12^ have reported promising lead compounds against the FMN riboswitch that demonstrate efficacy in animal models. Another system of therapeutic interest^13^ is the cobalamin (Cbl) riboswitch,^14^ which is broadly distributed across bacteria.^15^ Different classes of Cbl riboswitches exhibit distinct binding preferences to forms of Cbl that differ only at their β-axial position (Fig. 1a).^16,17^ The biological forms of Cbl are 5′-deoxyadenosylCbl (AdoCbl) and methylCbl (MeCbl, **1**),^13^ but standard forms include the photolysis^18^ product hydroxoCbl (OHCbl **2**) and the photostable^19^ cyanoCbl (CNCbl, **3**) (Fig. 1a).

We recently identified several β-axial derivatives **4–7** that bind the *env8* MeCbl-selective riboswitch^20^ and regulate in-cell function (Fig. 1b,c).^21^ This was a surprising result given that these derivatives host bulky β-axial moieties (Fig. 1c) that present a significant steric problem within the binding pocket (**Supplementary Fig. 1a**). Chemical probing suggests that recognition of the higher affinity **4** and **7** involves the displacement of an adenosine (A20) from the RNA core (**Supplementary Fig. 1b**), which is likely replaced by the β-axial group.^21^ Displacement of A20 would yield a cryptic binding site distinct from the displacement of an adjacent adenosine by the 5′-deoxyadenosyl moiety observed in AdoCbl-selective riboswitches (**Supplementary Fig. 1c**).^20^ Our preliminary data suggest that the cryptic binding of **4** and **7** may explain their increased affinity and that chemical modifications to the β-axial group have robust effects on RNA binding.^21^

In this work, we synthesized an expanded library of Cbl derivatives that host systematically varied β-axial moieties (Fig. 1d,e) and employed a set of biochemical and cell-based assays to quantify their RNA binding affinities and regulatory activities. These data were used in a predictive modeling platform to determine the ligand properties associated with tight affinity and strong regulatory activity. As a complementary approach, we used structure-based design to unmask the cryptic β-axial binding pocket and discover lead compounds with affinity exceeding the native ligand. This structure-informed approach also enabled the identification of novel cryptic binders that are chemically distinct from Cbl. Collectively, our work outlines the molecular determinants of specific and high affinity base displacement RNA binding modes, which can guide future efforts to target these common RNA interactions.^22–25^

## Results

### Small molecule library design and synthesis

Our Cbl derivative library was synthesized using a previously established reduction-free method^26,27^ where the cyano group at the β-axial position of CNCbl is replaced by the R -group of a variable alkyne (Fig. 1d). While this approach enabled the synthesis of a diverse assortment of photostable β-axial modified Cbls **8–44** (Fig. 1e), reactions with alkynes containing amines or protonated nitrogen atoms were unsuccessful (**Supplementary Fig. 2**). The logic of our library construction was to diversify around lead compounds **4** and **7**. This strategy enabled specific medicinal chemistry questions to be addressed with QSAR studies. Within this framework, we can determine which chemical features modulate RNA binding and function.

Given that the majority of RNA-binding ligands are aromatic systems capable of π-stacking,^28–30^ many RNA-targeting lead compounds suffer from limited solubility, low affinity, and/or lack of specificity, limiting the scope and utility of the downstream medicinal chemistry that is required for successful hit-to-lead discovery campaigns.^1–3^ In our approach (**Supplementary Fig. 3a**), Cbl functions as a soluble “host” of a variable chemical “guest” that interacts with the *env8* RNA target in the minor groove. This target-guest interaction is anchored by contacts from the host corrin ring and a-axial group (Fig. 1a) to project the variable β-axial moiety to the same spatial location within the cryptic site. Thus, our model system enables a detailed and systematic approach to determine which chemical features confer high affinity base displacement RNA binding modes, which is common throughout RNA biology^22–25^ (**Supplementary Fig. 3b**).

### Cbl derivatives productively bind env8

**Figure 2:**
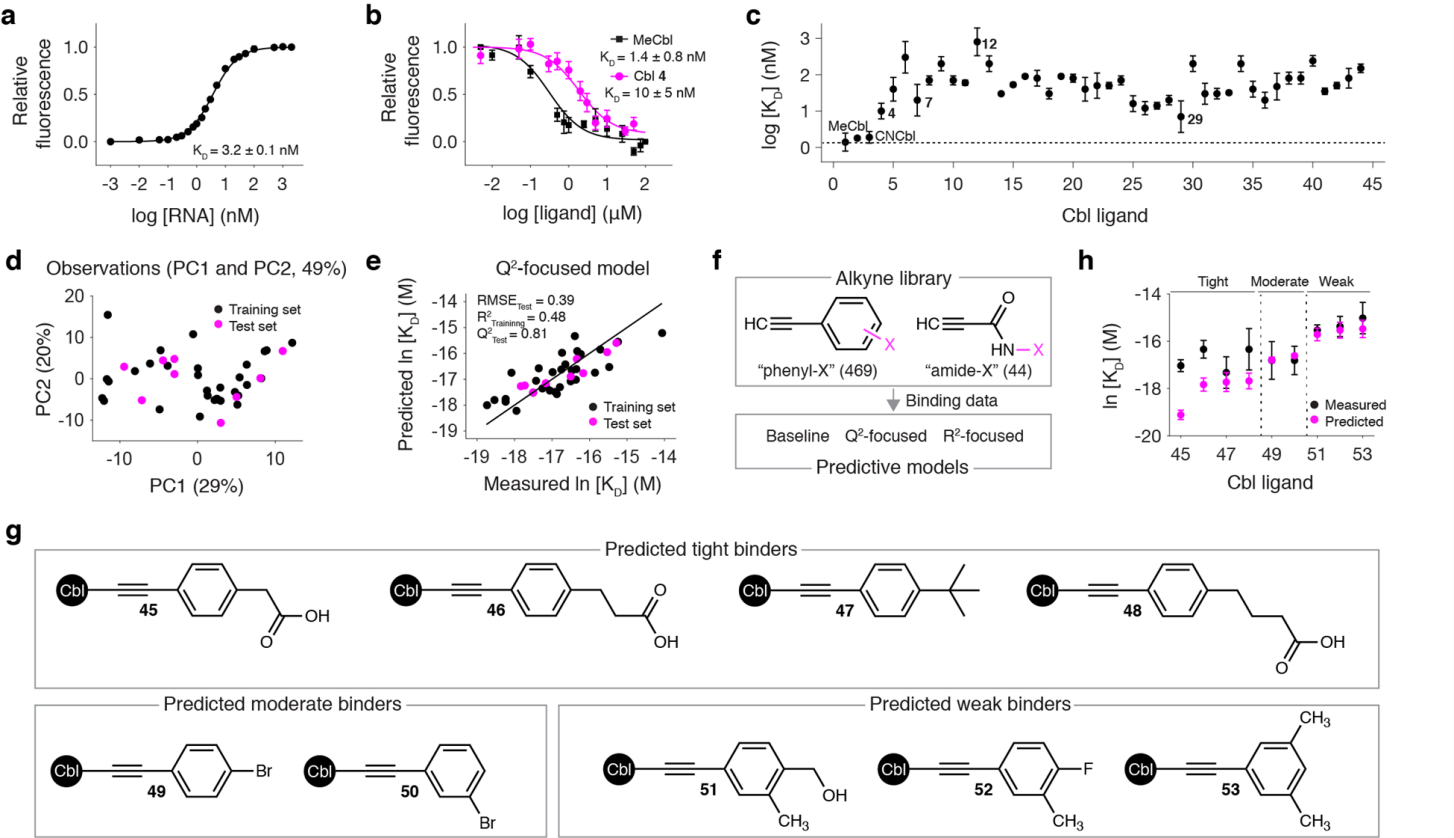
Measuring and predicting RNA binding affinity. **a,** Binding curve of the fluorescence induction of CNCbl-5×PEG-ATTO590 binding to *env8*. **b**, Representative displacement binding curve for MeCbl and Cbl **4. c**, Log-transformed K_D_ values for **1–44**. In **a-c**, mean and s.d. from independent experiments (*n* = 3–5) are shown. **d**, Locations of the training and test set from the Q^2^-focused modeling in two-dimensional chemical space constructed from PC1 and PC2 of the whole data set. **e**, Measured In [K_D_] values plotted with the value predicted by the Q^2^-focused model. **f,** Overview of our lead compound screen from a 513-compound alkyne library using our binding-based models. **g**, Simplified chemical structures of representative derivatives that our binding-based models predict to be tight (**45–48**), moderate (**49** and **50**), and weak (**51–53**) binders. **h**, Measured ln [K_D_] values plotted with the value predicted by our binding-based models. The experimental data are represented as mean and s.d. from independent experiments (*n* = 3–5) and the predicted data are shown as the mean and s.d. from independent predictions (*n* = 3) from our three models (baseline, R^2^-focused, and Q^2^-focused).

To quantify the binding of our library to *env8*, we used a fluorophore-conjugated CNCbI probe (CNCbI-5×PEG-ATTO590^31^) that undergoes fluorescence induction upon *env8* binding and competed it off with ligands in our derivative library.^21^ Titration of *env8* into the probe gave a K_D_ of 3.2 ± 0.1nM (Fig. 2a) and competitive titrations of MeCbl and Cbl **4** into the *env8*-CNCbl-5×PEG-ATTO590 complex yielded K_D_ values of 1.4 ± 0.8 nM and 10 ± 5nM, respectively (Fig. 2b). Using this fluorescence displacement assay, we measured the K_D_ values for Cbls **1–44**. All ligands bound *env8* with sub-micromolar affinity (Fig. 2c). The tightest binding derivative was **29** (7 ± 7 nM) while **12** (800 ± 700nM) was the weakest. Importantly, **29** functions as a binding lead with an affinity that exceeds **4** and **7** and is comparable (P = 0.19) with the native MeCbl.

### QSAR analysis of RNA binding data

To reveal chemical trends within our binding data, we employed a QSAR analysis. We quantified the “distance from the alkyne” of our *ortho-*, *meta-*, and *para*-substituted “phenyl-F” (**9–11**) and “phenyl-CH3” (**12–14**) derivatives and compared this parameter against their K_D_ values. We observed a strong relationship (R^2^ = 0.97) for the smaller substituents and an even stronger relationship (R^2^ = 0.99) for the larger groups (**Extended Data** Fig. 1a,b), suggesting steric limitations within the cryptic site. We also quantified the electron donating and withdrawing strength or Van der Waals (VdW) volume of the chemical groups in the *para*-substituted phenyls (**4, 5, 8, 11, 14**, and **17–20**) and compared these parameters against their K_D_ values. This analysis revealed that phenyl ring electronics (R^2^ = 0.10) and *para*-position sterics (R^2^ = 0.01) both fail to explain *env8* binding (**Extended Data** Fig. 1c-e).

Additional QSAR trends emerge from our heterocyclic Cbls (**21–26**) (**Extended Data** Fig. 1f). Comparing the affinities of phenyl **8** (70 ± 20 nM) and pyrimidine **24** (70 ± 20nM) suggest that heterocycles have no advantage over their hydrocarbon counterpart. Data from thiophene **21** (40 ± 30nM), thiazole 22 (50 ± 40 nM), and furan **23** (50 ± 10 nM) demonstrate that five-membered heterocycles bind just as well as six-membered ones. Finally, the increased affinity for our two-ring heterocycles **25** (16 ± 8nM) and **26** (12 ± 5 nM) suggests that two rings are preferred, which is consistent with our hypothesis that the β-axial group π-stacks within the cryptic site. This notion is supported by the binding data of our two-ring containing Cbls **27–29**, which all have affinities under 20 nM (**Extended Data** Fig. 1g). Even the three- (**31**, 30 ± 20 nM) and four-ring (**32**, 30 ± 10nM) containing derivatives bind with high affinity (**Extended Data** Fig. 1g).

To analyze these chemical trends in greater detail, we employed a multivariate analysis. Binding data were clustered into tight, moderate, and weak binders (**Extended Data** Fig. 2a), and 20 standard chemoinformatic parameters^32–35^ (**Supplementary Table 1**) were calculated for the β-axial group of Cbls **4**, **5**, and **7–44**. These descriptors were used in a linear discriminant analysis (LDA)^34,35^ with our binding data. When viewed along the first two principal components (PC1 and PC2), each binding cluster occupies unique regions in chemical space (**Extended Data** Fig. 2b). The LDA loading plot provides qualitative trends for the molecular determinants of each cluster. For example, tight binders have more aromatic rings, moderate binders have more hydrogen bond donors, and weak binders have more accessible surface area (**Extended Data** Fig. 2c).

### QSAR model-based screening of potential binding leads

Motivated by the LDA-identified trends, we sought to determine if the chemical identity of the β-axial group could be used to predict *env8* binding affinity. An expanded set of 347 physiochemical descriptors were calculated for the β-axial group of Cbls **4**, **5**, and **7–44** and modeled against their natural log-transformed K_D_ data using a least absolute shrinkage and selection operator (lasso)-multiple linear regression (MLR) strategy.^36^ When using all data in training, we obtained a baseline model that predicted our binding data with good accuracy (R^2^ = 0.71) using six descriptors (**Extended Data** Fig. 3a,b), which are listed along with their physical meaning in **Supplementary Table 2**. When using a stratified data split (Fig. 2d and **Extended Data** Fig. 3c) and implementing our lasso-MLR strategy, we constructed new models that prioritized either the predictive ability on the test set (Q^2^-focused) or the overall fit of the training set (R^2^-focused). The Q^2^-focused (R^2^_Training_ = 0.48, Q^2^_Test_ = 0.81) and R^2^-focused (R^2^_Training_ = 0.65, Q^2^_Test_ = 0.60) models showed a similar level of performance (Fig. 2e and **Extended Data** Fig. 3d).

We then used our binding-based models to screen for high affinity β-axial groups among a 513-compound alkyne library, which included modified-phenyl (“phenyl-X”) and aliphatic (“amide-X”) alkynes (Fig. 2f) that are compatible with our Cbl synthesis (Fig. 1d) and representative of our initial library (Fig. 1e). Among these compounds, 184 were predicted to have tighter affinities than our current binding lead **29** (**Extended Data** Fig. 3e). However, the majority of these small molecules were synthetically inaccessible using the non-reductive method^26,27^ (**Extended Data** Fig. 3f). These considerations left us with four remaining leads, which were synthesized into the corresponding Cbls **45–48** along with compounds that were predicted to be moderate (**49** and **50**) and weak (**51–53**) binders (Fig. 2 g). We then used our fluorescence displacement assay to quantify the binding of these derivatives to *env8*. The experimentally derived ln [K_D_] data agreed well with our model-based predictions for the moderate and weak binders but deviated significantly for the predicted leads **45** and **46** (Fig. 2h). One explanation for this observation is the sparseness of tight binders in our dataset (8 of 44, 18%). Synthetic routes that access chemical groups facilitating high affinity RNA binding interactions (e.g., amino or other positively charged groups^28–30^ could address this issue.

### Cbl derivatives promote RNA regulatory activity

**Figure 3:**
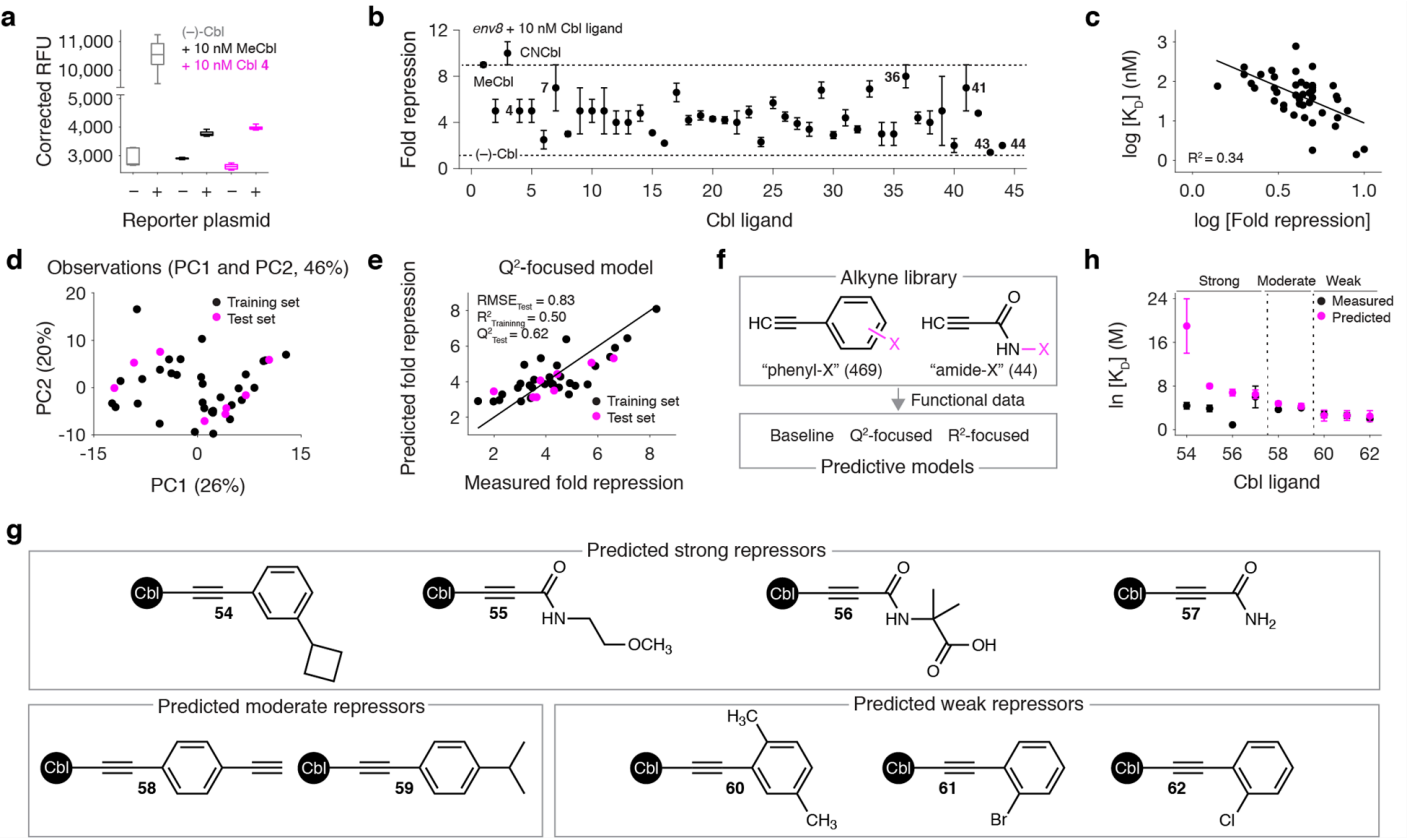
Measuring and predicting RNA regulatory activity. **a**, Box-and-whisker plot of the OD_600_-corrected relative fluorescence units (RFU) of *E. coli* cells with and without reporter plasmid (*env8*-GFPuv) and ligand (MeCbl and Cbl **4**) added. **b**, Fold repression (defined as the ratio of “(−)-Cbl RFU” and “(+)-Cbl RFU” values )^37^ for our *env8* reporter system in the presence of Cbl **1–44**. Mean and s.d. from biological replicates (*n* = 4) are shown. **c**, Plot comparing the log-transformed fold repression and K_D_ data. **d**, Locations of the training and test set from the Q^2^-focused modeling in two-dimensional chemical space constructed from PC1 and PC2 of the whole data set. **e**, Measured fold repression values plotted with the value predicted by the Q^2^-focused model. **f**, Overview of our lead compound screen from a 513-compound alkyne library using our function-based models. **g**, Simplified chemical structures of representative derivatives that our function-based models predict to be strong (**54–57**), moderate (**58** and **59**), and weak (**60–62**) repressors. **h**, Measured fold repression values plotted with the value predicted by our function-based models. The experimental data are represented as mean and s.d. from biological replicates (*n* = 4) and the predicted data are shown as the mean and s.d. from independent predictions (*n* = 3) from our three models (i.e., baseline, R^2^-focused, and Q^2^-focused).

Given that all the derivatives in our library bind *env8*, we wanted to determine whether they drive RNA regulatory activity in a cellular environment. We employed a previously established^37^ cell-based assay in which *env8* was placed upstream of GFPuv, whose expression is repressed by Cbl-dependent occlusion of the ribosome-binding site. In the absence of Cbl, we detected high fluorescence, indicative of GFPuv expression (Fig. 3a). Conversely, in the presence of 10 nM of MeCbl or Cbl **4**, we observed attenuated fluorescence, demonstrating that these ligands promote *env8* repression of GFPuv expression in *E. coli* (Fig. 3a).

To quantify the extent to which each derivative regulates *env8* function, we calculated the fold repression^37^ for Cbls **1–44**. While all ligands were able to regulate *env8* function to some extent, the repression was highly variable. The strongest repressors were **36** (8 ± 1) and **41** (7 ± 2) while **43** (1.4 ± 0.1) and **44** (2.0 ± 0.1) were the weakest (Fig. 3b). Importantly, **36** and **41** are new functional leads with fold repression values that exceed **4** and **7** and are comparable (**36**, *P* = 0.31 and **41**, *P* = 0.19) with the native MeCbl (9.0 ± 0.2).

These data enable the exploration of the relationship between derivative binding and regulatory activity. In general, while many tight binding derivatives are also strong repressors and vice versa, this was not always true (R^2^ = 0.34) (Fig. 3c). This fact reflects the complex nature of promoting a ligand-induced regulatory response, which involves Cbl cellular import, β-axial repair by endogenous enzymes, and *env8-*Cbl binding within the appropriate time scale of a co-transcriptional process.^37^ To investigate this further, we carried out growth experiments in ΔMetE cells, which lack the Cbl-independent methionine synthase MetE.^38^ In the absence of methionine, these cells must use Cbl for one carbon metabolism, making MeCbl essential for growth.^38^ In the presence of 10 nM derivative, both weak (Cbl **44**) and strong (Cbl **36**) repressors support ΔMetE growth with the same doubling time as CNCbl, suggesting that these ligands are imported and repaired to some extent (**Extended Data** Fig. 4). However, the fact that repression is variable among our derivatives in wild-type *E. coli* cells suggests that these compounds exist predominantly in their unrepaired, β-axial derivatized state. A confounding factor in comparing the binding and functional data is potentially variable levels of derivative metabolism within the cell.

### QSAR model-based screening of potential functional leads

While individual QSAR trends were less pronounced within our functional data (**Supplementary Fig. 4**), an LDA using the same set of chemoinformatic parameters^32–35^ (**Supplementary Table 1**) demonstrated that functional clusters (**Extended Data** Fig. 5a) occupy unique regions in chemical space (**Extended Data** Fig. 5b). The LDA loading plot suggests that strong repressors have more hydrogen bond donors, moderate repressors have more aromatic rings, and weak repressors have more sp^3^ centers (**Extended Data Fig. 5c**). These trends motivated us to build QSAR models using the same lasso-MLR workflow^36^ described above, except using our repression data as the dependent variable. We obtained baseline, Q^2^-focused, and R^2^-focused models that predict the functional data reasonably well with four to six descriptors (Fig. 3d,e and **Extended Data** Fig. 6a-d), which are listed in **Supplementary Table 3**.

We then used our function-based models to screen for strong repressive β-axial groups among the same alkyne library (Fig. 3f). Although 151 small molecules were predicted to have stronger repression than our current functional lead **36** (**Extended Data** Fig. 6e), many of these leads were unavailable for the same reasons described above (**Extended Data** Fig. 6f). We identified four available predicted leads, which were synthesized into the corresponding Cbl **54–57** along with compounds that were predicted to be moderate (**58** and **59**) and weak (**60–62**) repressors (Fig. 3g). The fold repression of **54–62** were then measured using our reporter assay. Much like our binding data, the experimentally derived repression data agreed well with our model-based predictions for the moderate and weak repressors but deviated significantly for the predicted functional leads **54–56** (Fig. 3h), which is likely explained by the confounding role of derivative metabolism on regulatory activity.

### env8 -Cbl co-crystal structures unmask cryptic binding site

**Figure 4:**
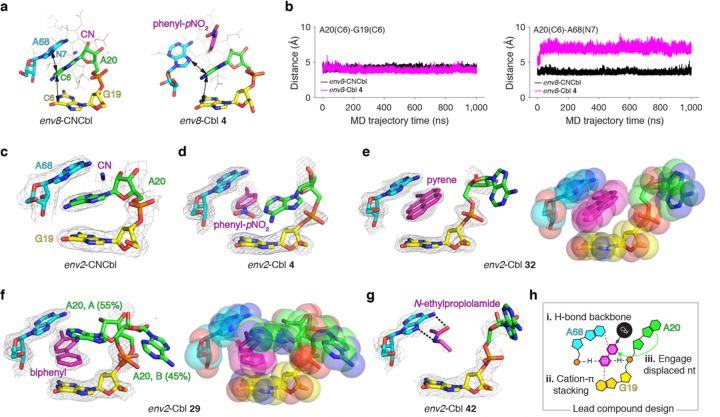
Co-crystal structures confirm A20 displacement and inform lead compound design. **a**, Representative MD starting states of the *env8*-CNCbl and *env8*-Cbl **4** complexes from one (of three) independent 1 μs trajectory. The A20(C6)-G19(C6) and A20(C6)-A68(N7) distances are shown as double arrows. In all structural representations, the binding pocket nucleotides G19 (yellow), A20 (green), and A68 (cyan) are numbered in reference to full-length *env8* and colored, the β-axial group is shown in magenta, and dashed lines represent proposed hydrogen bonds. **b**, The average A20(C6)-G19(C6) (left) and A20(C6)-A68(N7) (right) distances measured over the course of three independent MD trajectories for both *env8-*CNCbl and *env8*-Cbl **4** complexes. **c**, Co-crystal structure of *env2*-CNCbl showing only the binding pocket nucleotides and the β-axial group. All mesh representations correspond to a simulated annealing 2F_o_-F_c_ map where A20 and the ligand were omitted from the model and are shown at 1 σ contour. **d**, Same as in c but for *env2*-Cbl 4. **e**, Same as in **c** but for *env2*-Cbl **32** (left) and also showing a Van der Waal sphere representation of the binding pocket (right). f, Same as in **e** but for *env2*-Cbl **29**. **g**, Same as in **c** but for *env2*-Cbl **42**. **h**, Schematic representation of design principles that emerge from our 11 RNA-ligand co-crystal structures.

As a complementary approach to QSAR modeling, we used structure-based design to investigate *env8-*ligand interactions. We carried out three independent 1 μs molecular dynamics (MD) simulations of *env8*-CNCbl and *env8*-Cbl **4** and measured the A20(C6)-G19(C6) and A20(C6)-A68(N7) distances to assess A20 π-stacking with neighboring nucleotides (Fig. 4a). Over the course of the MD trajectories, the A20(C6)-G19(C6) distances were identical among both complexes, while **4** did induce slight increases in A20(C6)-A68(N7) distances (Fig. 4b). However, these changes were not a result of A20 displacement but were caused by the β-axial phenyl-*p*NO_2_ group intercalating between A20 and A68 (**Supplementary Fig. 5**), which is inconsistent with previous chemical probing data (**Supplementary Fig. 1b**).^21^

The limitations of our MD analysis motivated us to employ X-ray crystallography. We determined co-crystal structures of the Cbl riboswitch aptamer domain in complex with CNCbl and **4**. However, we only obtained diffraction-quality crystals using the closely related *env2* RNA, which shares 95% sequence identity with *env8* (**Extended Data Fig. 7a**). Given that *env2* shows near-identical ligand binding and regulatory activity as *env8* (**Extended Data Fig. 7b,c**), all structural information can be translated to *env8*. The *env2*-CNCbl structure shows A20 π-stacked with G19 and A68 (Fig. 4c) with strong agreement to the *env8*-OHCbl structure^20^ (RMSD = 0.45 Å) (**Extended Data Fig. 7d**). The *env2*-Cbl **4** structure, on the other hand, reveals that A20 undergoes base displacement from the RNA core toward the major groove and that the β-axial phenyl ring π-stacks with A68 (Fig. 4d), unmasking the cryptic binding pocket in agreement with our hypothesis.^21^ We also determined the structure of *env2* in the apo state, which shows a near-identical (RMSD =0.39 Å) binding pocket to our *env2*-CNCbl structure (**Extended Data Fig. 8a**), demonstrating that CNCbl interacts with a pre-organized binding pocket. Notably, the *env2*-Cbl **4** structure reinforces the inability of the MD simulation to access base displacement of nucleotides.

To gain further insight into cryptic β-axial pocket, we determined nine additional *env2*-ligand co-crystal structures (**Extended Data Fig. 8b-j**). The structure of *env2*-Cbl **32** with its bulky pyrene β-axial group showcases that base displacement provides a lot of room (>340 Å^3^) in the binding pocket (Fig. 4e). With its four phenyl rings, pyrene π-stacks well with A68 but is unable to optimize π-stacking with G19 (Fig. 4e). In contrast, the structure of *env2*-Cbl **29** demonstrates how the biphenyl β-axial group maximizes π-stacking with both G19 and A68 via rotation of the two rings relative to one another (Fig. 4f). Interestingly, our data support the fitting of two conformations of A20, with the more populated (55%) conformer partially engaged within the RNA core (Fig. 4f), providing a means to offset the energetic penalty of A20 displacement. It is important to note that π-stacking is not the only binding mode available within the cryptic site. The structure of *env2*-Cbl **42** suggests that two hydrogen bonds to A68 provide comparable affinity (50 ± 10nM) with a π-stacking interaction (e.g., **8**, 70 ± 20nM) (Fig. 4 g). Together, our structural data suggest a set of design principles to develop new lead compounds: (1) hydrogen-bonding to the phosphate backbone, (2) cation-π-stacking, and (3) engagement of A20 (Fig. 4h).

### Structure-based design identifies Cbls with affinity exceeding the native ligand

**Figure 5:**
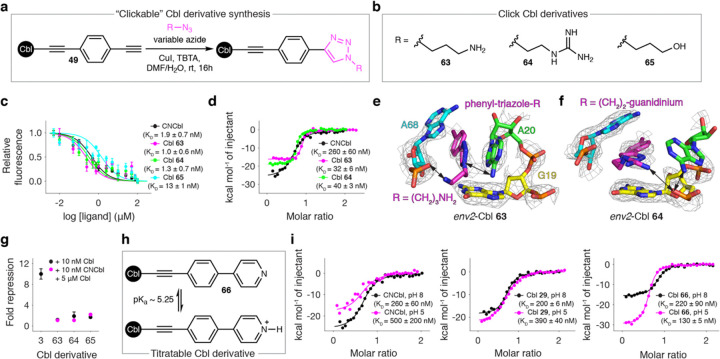
Structure-based design of lead compounds with affinity exceeding the native ligand. **a,** Modified click reaction^26^ to make Cbl derivatives from **58** using a variable azide. **b**, Simplified chemical structures of Cbls **63–65** that were made to systematically test our design principles. c, Fluorescence displacement binding curve for CNCbl and **63–65**. K_D_ values are reported as mean and s.d. from independent experiments (*n* = 3–5). **d**, To verify the near-picomolar binding of **63** and **64**, we used ITC with the *env8* aptamer domain. Given that this RNA shows weaker ligand binding than the full-length *env8*,^20^ it is in the perfect regime for ITC. Representative ITC binding isotherm for the *env8* aptamer domain with CNCbI, **63**, and **64**. **e**, Co-crystal structure of *env2*-Cbl **63** showing only the binding pocket nucleotides and the β-axial group. Proposed hydrogen bonds, which are weakly supported by the electron density, are shown as double arrows. All mesh representations correspond to a simulated annealing 2F_o_-F_c_ map where A20 and the ligand were omitted from the model and are shown at 1 σ contour. **f**, Same as in **e** but for *env2-*Cbl **64. g**, Fold repression for our reporter system in the presence of CNCbl and **63–65** and for cells with CNCbl and excess amounts of 63–65 added. Mean and s.d. from biological replicates (*n* = 4) are shown. **h**, Simplified chemical structure of the titratable pyridine Cbl **66**. **i,** Representative ITC binding isotherms for *env8* aptamer domain with CNCbI, **29**, and **66** at pH 8 and 5. For all ITC data, K_D_ values are reported as mean and s.d. from independent experiments (*n* = 3).

In order to explore design principles (1) and (2), we adopted new chemistry using a click reaction of Cbl **58** with a variable azide^26^ to form a biphenyl-like scaffold where the second ring is an R-group harboring triazole (Fig. 5a). We synthesized Cbls **63–65** to install amino-, guanidinium-, and hydroxyl-substituted triazoles, respectively (Fig. 5b). We then used our fluorescence displacement assay to quantify the binding of these derivatives to *env8* and discovered that **63**(1.0 ± 0.6nM, *P* = 0.62) and **64** (1.3 ± 0.7 nM, *P* = 0.92) have comparable affinity to CNCbl (Fig. 5c). However, these interactions approach the accuracy limit of our binding assay. To confirm these results, we used isothermal titration calorimetry (ITC) with the *env8* aptamer domain, which lacks an element of RNA structure that interacts with the α-axial face of the Cbl and thereby lowering the observed affinity. These data revealed significantly tighter affinity to **63** (32 ± 6 nM, *P* = 0.02) and **64** (40 ± 3nM, *P* = 0.02) as compared to CNCbI (260 ± 60 nM) (Fig. 5d).

To understand how these new lead compounds bind *env8*, we determined co-crystal structures of *env2*-Cbl **63** and *env2*-Cbl **64**. While weakly supported by the electron density, both structures suggest that the amino (Fig. 5e) or guanidinium (Fig. 5f) groups likely electrostatically interact with backbone phosphate of G19 (*env2*-Cbl **64**) or A68 (*env2*-Cbl **63**). However, our *env2*-Cbl **63** structure unambiguously demonstrates that the terminal triazole ring twists in order to π-stack with A20 (Fig. 5e), again suggesting that rotatable bonds are beneficial for optimizing the π-stacking network. This is the first evidence of strong engagement of A20 in any of our structures. Notably, when π-stacked with the ligand, A20 adopts a perpendicular orientation relative to G19, a highly unusual mode of base-base interaction in nucleic acid.

Despite their high affinity, when used in our reporter assay, Cbls **63–65** confer minimal repression (Fig. 5 g). To investigate this further, we repeated our growth experiments in ΔMetE cells. In the presence of 10 nM derivative, **63–65** support ΔMetE growth with the same doubling time as CNCbl, suggesting that these ligands are imported and repaired to some extent (**Extended Data Fig. 9a,b**). However, unlike **36** and **44**, elevated concentrations of **63–65** prevented growth of ΔMetE cells (**Extended Data Fig. 9c,d**), suggesting that these derivatives antagonize some essential aspect of Cbl metabolism in *E. coli*.^39^ To fully assess how **63–65** affect regulatory activity, we repeated our reporter assay in wild-type *E. coli*, which grow normally in the presence of high Cbl concentrations (**Extended Data Fig. 9e**). Adding excess amounts of **63–65** to cells containing 10 nM CNCbI resulted in loss of CNCbl-induced repression, confirming that these derivatives are antagonists of *env8* function (Fig. 5g).

To further explore design principle (2), we synthesized the pyridine Cbl **66** to introduce a conditional positive charge (Fig. 5h). Under the conditions of our binding experiments (pH 8), **66** should behave exactly like its non-pyridine counterpart **a**. However, at a pH below its predicted pK_29_ (~ 5.25), **66** should be charged and we would expect an increase in affinity that is absent in **29**. To test this hypothesis, we carried out ITC measurements at pH 8 and 5 with the *env8* aptamer domain and CNCbl **29**, and **66**. The non-titratable CNCbl and **29** showed a ~ two-fold reduction in binding affinity at reduced pH, whereas the affinity of **66** increased 1.7-fold (Fig. 5i), in agreement with our hypothesis. These data provide proof-of-principle that installing pyridines, whose pK_a_ can be lowered by interaction with RNA, is an important design principle that leverages the increased affinity of cation-π interactions without violating Lipinski’s rules.^40,41^

### Structure-informed screen identifies cryptic binders of env8 divorced from Cbl

**Figure 6:**
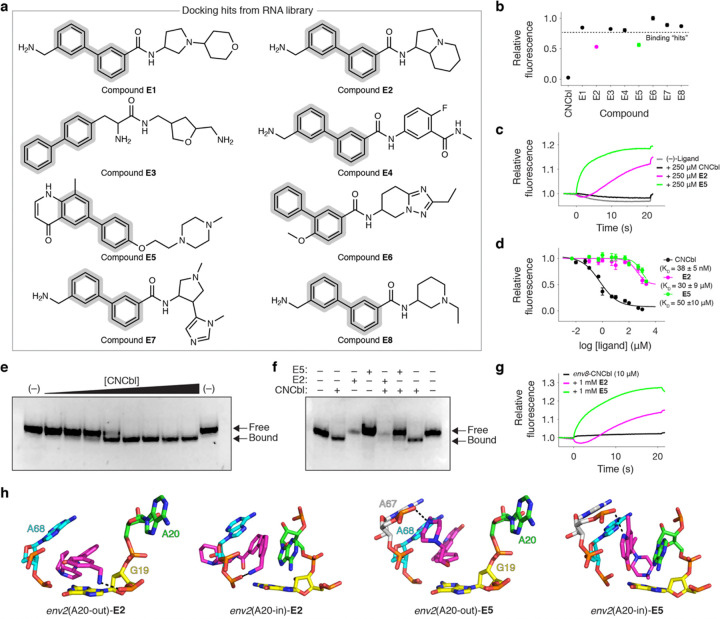
Structure-informed screen identifies cryptic binders of *env8* divorced from Cbl. **a,** Chemical structure of the eight hits that emerged from our structure-informed docking, with their biphenyl scaffold highlighted in gray. **b,** TO-based displacement assay to identify *env8* binding compounds that induce fluorescence attenuation. This was used as an orthogonal binding experiment because we observed ligand-induced fluorescence induction of CNCbl-5×PEG-ATTO590. **c**, Representative MST trace of fluorescently labeled *env8* alone and in the presence of CNCbl, **E2**, and **E5**. All ligands induced an observable change to the MST trace of *env8* suggestive of binding. **d**, TO-displacement binding curve for CNCbl, **E2,** and **E5**. K_D_ values are reported as mean and s.d. from independent experiments (*n* = 3). **e,** Native polyacrylamide gel showing a gradual shift from a slower migrating, extended conformation to a faster migrating, kissing-loop conformation of *env8* upon titration of CNCbI (0.01–10 μM). **f**, Same as in **e** but after addition of CNCbI, **E2,** and/or **E5** to demonstrate competitive binding of **E2** and **E5. g,** Representative MST trace from a competition experiment where *env8*-CNCbl was monitored with and without the addition of excess amounts of **E2** or **E5**. Both ligands induced an observable change to the MST trace of *env8*-CNCbl suggestive of competitive binding. **h**, Top-ranked docking poses of **E2** and **E5** to *env2* in the A20-out and A20-in state.

Our structural biology efforts unmasked the cryptic β-axial binding pocket within *env8*, which allows us to explore whether small molecules divorced from a Cbl host can also target this site. To address this issue, we carried out a high-throughput computational screen using a 28,000-compound RNA-focused library. These compounds were docked against *env2* in two conformations: one in which A20 was displaced toward the major groove (A20-out, e.g., *env2*-Cbl **29**) and one where A20 was engaged in the RNA core (A20-in, e.g., *env2*-Cbl 63) (**Supplementary Fig. 6**). To leverage our structural data, we specified that docking hits contain the biphenyl-like scaffold and identified eight potential lead compounds (Fig. 6a).

To survey the binding of these ligands to *env8*, we adopted a thiazole orange (TO) displacement assay (**Supplementary Fig. 7a**).^42^ Upon addition of **E1-E8**, **E2** and **E5** led to sufficient (> 25%) displacement of TO indicative of binding (Fig. 6b). This conclusion was confirmed with microscale thermophoresis (MST) (Fig. 6c). Competitive titrations of CNCbI, **E2**, and **E5** into the *env8*-TO complex yielded K_D_ values of 38 ± 5nM, 34 ± 9 μM, and 50 ± 10 μM, respectively (Fig. 6d). The affinity of CNCbl agrees well with data obtained from ITC (38 ± 8nM) (**Supplementary Fig. 7b,c**), supporting the use of this method to quantify ligand binding to *env8*.

To verify that **E2** and **E5** target the Cbl binding pocket, we employed an electrophoresis mobility shift assay (EMSA) that monitors the ability of *env8*to adopt a kissing-loop conformation in the presence of Cbl,^37^ which migrates faster than the unbound RNA (Fig. 6e). Given that the kissing-loop is facilitated by interactions with the α-axial face of the Cbl corrin ring,^37^ **E2** and **E5** are not expected to induce this conformational change. Thus, if these ligands competitively bind the same site within *env8*, then adding **E**2 or **E5** to *env 8* in the presence of CNCbl should revert the RNA back to the slower migrating species. When excess amounts of **E2** or **E5** were added to an *env8*-CNCbI complex both ligands prevented kissing-loop formation, suggestive of competitive binding (Fig. 6f). While this phenomenon was unambiguous for **E5**, addition of **E2** reproducibly led to weaker gel staining (Fig. 6f). We therefore confirmed the competitive binding of both ligands with MST (Fig. 6g). These data support the top-ranked docking poses of **E2** and **E5** which show their biphenyl-like scaffolds maximizing π-stacking to G19 and A68 within the cryptic β-axial binding pocket and making hydrogen bonds to the phosphate backbone (Fig. 6h).

## Discussion

Targeting RNA with small molecules is an emerging field hindered by an incomplete understanding of the basic principles governing RNA-ligand interactions.^1–3^ To address this knowledge gap, we explored the chemical features promoting specific and high affinity base displacement RNA binding interactions using the Cbl riboswitch. Despite limitations of our QSAR chemoinformatic modeling, structure-based design successfully identified lead compounds with affinities exceeding the native ligand. All leads share a biphenyl-like structure, which has been previously identified as a privileged RNA-binding scaffold.^28–30,43^ Our data demonstrate that twisting around a rotatable bond confers the ability to optimize the π-stacking network within the binding pocket. Thus, our results not only reinforce our understanding of RNA chemical space but yield new structural insights into how these scaffolds interact with RNA.

Across all newly reported RNA-ligand co-crystal structures, binding pocket nucleotides G19 and A68 are in near-identical positions, indicating that structural plasticity is conferred solely by the displacement of A20, providing a large (> 340 Å^3^) cryptic site that accommodates the β-axial group of all derivatives. One mechanism to explain this phenomenon is ligand docking into a rigid RNA to induce A20 displacement; however, large, rigid β-axial groups likely prevent the corrin ring from productively engaging the RNA. A more likely explanation is that A20 undergoes transient excursions from the RNA core to form a binding-competent state, a motion that is well documented in nucleic acids and consistent with the time scale of ligand binding.^44^ The energetic penalty of A20 displacement to expose the cryptic pocket is offset to varying degrees by each β-axial group, reflecting different derivative binding affinities. This illustrates that a consideration of local binding pocket dynamics is an essential feature to unmask additional RNA cryptic sites,^45,46^ which will facilitate the development of new chemical probes of RNA function and therapeutics to treat RNA-mediated disease. However, our work cautions against using a purely computational approach, as MD simulations could not account for the nuances of base displacement within *env8*. Advances in machine-learning^47^ and artificial intelligence^48^ methods may address these limitations in the future.

Our work also showcases that bifunctional host-guest systems are a valuable approach to targeting RNA. Our soluble Cbl host enabled access to chemical space that is typically intractable to robust medicinal chemistry. Exploration of general host-small molecule conjugates is therefore of critical importance. Nucleic acid-based hosts are an attractive candidate because they can solubilize the attached ligand, deliver it to RNA in a sequence specific manner, and contribute binding energy that can turn modest and/or non-specific binders to ligands with high affinity and specificity. For example, TO has been conjugated to a peptide nucleic acid (PNA) host to monitor double-stranded RNA formation.^49^ PNA hosts have also been shown to rescue the binding of small molecules that have no affinity for RNA by themselves.^50^ This conceptual framework is similar to the widespread use of bifunctional/multivalent small molecules in protein targeting,^51^ which has recently seen parallels in RNA-targeting strategies (e.g., RiboTAC^43^ and RiboSNAP^52^). We believe that a host-guest approach is generalizable and will enable detailed chemical and structural analysis of underexplored chemical space to better understand RNA-small molecule interactions.

## Methods

### Synthesis of Cbl derivatives

Cbl derivatives **4–66** were synthesized according to previously described procedures.^26,27^ See the **Supplementary Information** for additional details.

### RNA preparation

All RNAs (full sequences shown in **Supplementary Table 4**) were prepared using DNA templates amplified by PCR, transcribed with T7 RNA polymerase, and purified using preparative denaturing polyacrylamide gel electrophoresis.^53^ Purified RNA was buffer exchanged and concentrated into Milli-Q H_2_O using centrifugal concentrators (Millipore-Sigma). Final RNA concentrations were calculated using absorbance at 260 nm and extinction coefficients determined from the summation of the individual bases. Prior to all binding experiments, RNA was heated at 95°C for 2 minutes, incubated on ice for 10 minutes, and then allowed to equilibrate to room temperature.

### Cbl fluorescence binding assays

Both direct CNCbl-5×PEG-ATTO590 fluorescence induction and displacement assays were performed as previously described.^21^ The K_D_ values for all Cbl derivatives to *env8* can be found in **Supplementary Table 5**.

### Cell-based reporter assay

These were conducted as previously reported^37^ with minor alterations. Firstly, plasmids harboring *env8-*GFPuv (Addgene #99831) were transformed into *E. coli* BW25113 cells. Second, 1 μl of a saturated overnight culture was added to 1 ml of CSB media supplemented with 100 μg/ml carbenicillin and 10 nM Cbl (unless otherwise noted) and grown to mid-log phase at 37°C. Third, from each biological replicate, 200 μl of cells were added to wells in a 96-well plate (Costar). The fold repression values^37^ for all Cbl derivatives can be found in **Supplementary Table 6**.

### ΔMetE growth assays

For growth experiments, 5 μl of a saturated overnight culture of ΔMetE *E. coli* cells (Keio collection, JW3805) was added to 5 ml of methionine-dropout CSB media supplemented with either 335 μM methionine or 10 nM Cbl and grown to mid-log phase at 37°C. From each biological replicate, 200 μl of cells were added to wells in a 96-well plate (Costar) and growth was monitored by measuring OD_600_ using a microplate reader (Tecan). For growth rate experiments, the same procedure was used except the cells were grown for 24 hours and the OD_600_ was measured at various time intervals. For titration experiments, the same procedure was used except variable amounts of methionine or Cbl was added to the media.

### Chemoinformatic linear discriminant (LDA) analysis

A set 20 standard chemoinformatic parameters ^32–35^ (**Supplementary Table 1**) were calculated for the β-axial group of Cbls **4, 5**, and **7–44** as previously described.^34,35^ All descriptors that were non-constant were then used in a LDA with our clustered binding or functional data (see **Extended Data** Figs. 2 and 5) using Discriminatory Analysis plugin from XLSTAT as previously described.^34,35^ Excel files with the calculated descriptors are available upon request.

### QSAR modeling

The β-axial group of Cbls **4**, **5**, and **7–44** were tuned to the correct protonation and tautomerization states using molecular operating environment (MOE, v2022.02, Chemical Computing Group) and each state was sent to conformational search as previously described.^36^ A total of 347 descriptors (all but x3D and protein descriptors) were calculated for each conformation in MOE, averaged using the Boltzmann-weighted equation, and filtered to remove multicollinear features as previously described.^36^ Then, a lasso-MLR strategy was used to model these descriptors (independent variable) against either the natural-log transformed K_D_ or fold repression data (dependent variables) in matlab (v2019a). Lasso was implemented with five-fold cross-validation to find an optimal lambda value in descriptor selection. MLR exhaustively searched all possible models from the lasso-selected descriptors (with a maximum number of descriptors set by Topliss rule^54^) with five-fold cross-validation to obtain the best model as defined by specified criteria. We constructed a baseline model without a data split that maximized the R^2^. We also used a stratified 80/20 data split to ensure that training and test sets had equivalent proportions of tight/strong, moderate, and weak binders/repressors and constructed models that prioritized either the predictive ability on the test set (Q^2^-focused) or the fit of the training set (R^2^-focused). Excel files with the calculated descriptors and matlab scripts for modeling are available upon request.

### Lead compound prediction

We used our binding- and function-based models to screen for high affinity β-axial groups among a curated Enamine alkyne library. These small molecules were selected by a substructure search (https://enaminestore.com/search) of modified-phenyl (phenyl-X) and aliphatic (amide-X) alkynes (precise structures used for this search are shown in Fig. 2f). This left us with a 513-compound library (469 phenyl-X and 44 amide-X). The binding- and function-based model-selected descriptors (**Extended Data** Figs. 3a and 6a) were calculated for the 513-compound library in MOE as described above. The predicted ln [K_D_] and fold repression values from baseline, Q^2^-focused, and R^2^-focused models were averaged and used to select tight, moderate, and weak binders (Cbls **45–53**) (Fig. 2g) and strong, moderate, and weak repressors (Cbls **54–62**) (Fig. 3g). Excel files with the calculated descriptors and SDF files from phenyl-X and alkyne-X substructure search are available upon request.

### Molecular dynamic (MD) simulations

All-atom MD simulations of *env8*-CNCbl and *env8*-Cbl **4** complexes were performed using the PMEMD module^55^ in AMBER22.^56^ CNCbl was manually built from OHCbl (PDB 4FRG^20^) in PyMol (v2.5.7, Schrödinger) and Cambridge Structural Database deposition 961228^27^ was used for Cbl **4**. Ligands were docked into *env8* by alignment to OHCbl from the *env8*-OHCbl structure (PDB 4FRG^20^). Each system was neutralized and solvated in a cubic simulation box with a 25 Å buffer of OPC water molecules^57^ and 150 mM NaCl to mimic physiological conditions. RNA was parameterized using the OL3 force field,^58^ incorporating the RNA.LJbb correction^59^ for improved backbone accuracy. Force field parameters for each ligand were generated using Antechamber with the General Amber Force Field. The metal center, including the cobalt ion, was parameterized using MCPB.py following established guidelines.^60^ Quantum mechanical calculations were performed with Gaussian16 to optimize the geometry and derive charges. The cobalt-carbon bond was explicitly defined in the MCPB.py input to ensure accurate representation of the ligand coordination environment.^60^ To improve simulation efficiency, hydrogen mass repartitioning was applied, allowing a 4 fs time step.^61^ Following system preparation, energy minimization, gradual heating to 300 K, and equilibration under constant pressure were conducted. Production simulations were performed in triplicate for each system under constant temperature (300 K) and pressure (1 atm) using the NPT ensemble, with each replicate running for at least 1 μs. Trajectory analysis and post-processing A20(C6)-G19(C6) and A20(C6)-A68(N7) distance calculations were carried out using Cpptraj.^62^

#### Crystallization of env2-ligand complexes

The *env2* aptamer domain RNA in complex with various Cbl derivatives was crystallized at 30°C using hanging drop vapor diffusion. The RNA-ligand complexes were prepared as solutions containing 250 μM RNA and 375–500 μM ligand in 1X TE buffer. For crystallization, drops containing 2 μl of RNA-ligand solution and 2 μl precipitant solution (40 mM sodium cacodylate pH7, 10% (w/v) 2-methyl-2,4-pentanediol (MPD), 12 mM spermine tetrahydrochloride, 80 mM KCl, and 20 mM MgCl_2_) were suspended above a reservoir solution of 500 μl of 35% MPD and grown for 24–96 hours. For cryoprotection, the crystals were soaked in a cryoprotectant (precipitant solution with MPD increased to 25%) for 2 min and flash frozen in liquid nitrogen. Some data were collected on a home-source Rigaku MicroMax-003 X-ray source with a Dectris Pilatus 200K detector, and then indexed, integrated, and scaled with HKL3000^63^ (**Supplementary Tables 7–10**). Other data were collected at the Advanced Light Source on a Dectris on the ALS Beamline 8.2.2. with a Dectris Pilatus3 2M detector, and then indexed, integrated, and scaled with XDS^64^ (**Supplementary Tables 7–10**). The crystals that yielded the *env2* apo structure were grown in the same solution described above but in the presence of compound **E5**.

### Structure determination and model refinement

For all structures, an initial electron density map was calculated using molecular replacement with Phaser^65^ in PHENIX^66^ using *env8* as a starting model (PDB 4FRG^20^) but with OHCbl and A20 removed to minimize model bias. All ligands were manually built from Cbl **4** (Cambridge Structural Database deposition 961228^27^) in PyMol (v2.5.7, Schrödinger) and restraint files were generated with eLBOW.^67^ After initial refinement, the ligand density was unambiguous, and ligands were added to the model with LigandFit.^68^ After another round of refinement, A20 was manually built into the model. The map and model were refined, and the solvent was built until a maximum agreement between the map and the model was reached. During this process, to mitigate the effect of model bias, multiple rounds of combined high temperature (5,000 K) simulated annealing in torsion space and maximum-likelihood refinement were performed.^69^ At the end of refinement, RNA geometry was corrected with ERRASER^.70^ A subset of the RNA-ligand co-crystal structures had sufficient resolution to warrant simulated annealing in Cartesian space and individual ADP refinement. A simulated annealing 2F_o_-F_c_ map where A20 and the ligand were omitted from the model was calculated for all structures to support the final placement of the ligand and A20. Models and associated structure factors have been deposited in the RCSB Protein Data Bank.

### Isothermal titration calorimetry (ITC)

RNA was exchanged into a 1X RNA binding buffer (50 mM 4-(2-hydroxyethyl)-1-piperazineethanesulfonic acid (HEPES) pH 8.0, 100 mM KCl, 10 mM NaCl, and 1 mM MgCl_2_) by successive washing in centrifugal concentrators (Millipore-Sigma). RNA was diluted up to a final concentration of 10 μM and titrated with ligand (dissolved in the flow-through of the final wash from buffer exchange) at 100 μM. All titrations were performed at 25°C using a MicroCal ITC200 and collected in triplicate. The data were fit to a single-site binding model using Origin 7 ITC software (MicroCal).

### Computational docking

The 28,000-RNA library from Enamine (https://enamine.net/compound-libraries/targeted-libraries/rna) was downloaded as an SDF file and imported into MOE (v2022.02, Chemical Computing Group) as a database file. The small molecules were tuned to the correct protonation and tautomerization states and each state was sent to conformational search^36^ as described above. RNA receptor preparation was carried out using the default Quick Prep protocol in MOE. Our *env2*-Cbl **29** (A20-out) and *env2*-Cbl **63** (A20-in) structures were used as representative receptors where A20 was displaced toward the major groove or engaged in the RNA core, respectively. To ensure that A20 was not accessible for A20-out docking, all A20 atoms were removed from the RNA receptor in MOE. Rigid receptor docking was carried out in a templated manner, where ligand search space started from the biphenyllike scaffold present in the co-crystal structures with **29** and **63**. For A20-in docking, the terminal triazole ring was rebuilt into a phenyl group, so that the scaffolds were identical in both RNA receptors. With this approach, only the 319 ligands with the required scaffold were docked and scored.

### Thiazole orange (TO) binding assay

For fluorescence induction titration experiments, 60 μl reactions containing TO (5 nM), 1X RNA binding buffer, and increasing amounts of *env8* were incubated in 384-well plates (Corning). Reactions were incubated for 15 minutes and TO fluorescence was monitored (490 ± 10 nm excitation, 530 ± 10 nm emission) using a CLARIOstarPLU microplate reader (BMG Labtech). For competitive fluorescence displacement assays, the same procedure was carried out except with a fixed amount of 2 μM RNA, 20 μM TO (in excess of its K_D_ to ensure RNA saturation), and 2 mM competing ligand. Displacement titrations were carried out in the same manner except using increasing amounts of **E2** or **E5**. All titrations were performed at room temperature and collected in triplicate. The data were fit to a single-site binding model with a HillSlope parameter and the K_D_ from the induction experiment was used to convert the IC_50_ from the competitive fluorescence displacement assays to K_D_ values, as previously described.^21^

### Electrophoresis mobility shift assay (EMSA)

To validate the CNCbl-induced *env8* conformational change, 20 μl reactions containing RNA (0.5 μM), 1X RNA binding buffer, and increasing amounts of CNCbl (0.1–10 μM) were incubated at 37°C for 15 minutes and then at 4°C for 15 additional minutes. Once equilibrated to 4°C, 15 μl of the samples were loaded onto a native 10% (19:1) polyacrylamide gel in 0.5 X THE buffer and 1 mM MgCl_2_ and run at constant 7 W for 3 hours at 4°C in 0.5 X THE buffer with1 mM MgCl_2_. For the competitive EMSA experiment, the same procedure was used except using a pre-bound *env8*-CNCbI complex (0.5 μM CNCbl) and adding 5 mM **E2** or **E5**. All gels were stained with ethidium bromide, imaged on a UV illuminator (Alpha Innotech), and processed with AlphaView software (AlphalmagerHP). Unmodified images of the gels shown in Fig. 6e,f and their replicates can be found in **Supplementary Fig. 8**.

### Microscale thermophoresis (MST)

For MST binding experiments, *env8* was 3’-end labeled with pCp-AF488 (NU-1706-AF488, Jena Bioscience). The 50 μl labeling reaction comprised 200–500 nmol RNA, 1 mM ATP, 10% (w/v) DMSO, 1 X T4 RNA ligase 1 buffer (#B0204S, New England Biolabs (NEB)), 15% (w/v) polyethylene glycol 8,000, 48 μM pCp-AF488, and 4 μl T4 RNA ligase 1 (#M0204L, NEB) and was incubated at 16°C for 18 hours. To monitor RNA-ligand binding, labeled *env8* (300 nmol) was incubated in 1X RNA binding buffer and 250 μM ligand for 15 minutes and transferred to capillaries (#MO-K022, NanoTemper). Alexafluor488 fluorescence was read on a Monolith NT. 115 (NanoTemper) instrument. Competitive MST experiments were carried out in the same manner except using a pre-bound *env8*-CNCbI complex (300 nmol RNA and 10 μM ligand) and adding 1 mM **E2** or **E5**.

## Data Availability

Atomic coordinates and structure factors have been deposited in the Protein Data Bank (PDB) (https://www.rcsb.org/) under accession numbers 9MFH (*env2* apo), 9E5H (*env2*-CNCbI), 9E5I (*env2*-Cbl **4**), 9E5J (*env2*-Cbl **5**), 9E5K (*env2*-Cbl **13**), 9E5L (*env2*-Cbl **26**), 9E5M (*env2*-Cbl **29**), 9E50 (*env2*-Cbl **32**), 9E5P (*env2*-Cbl **33**), 9E5Q (*env2*-Cbl 3**6**), 9ELR (*env2*-Cbl **37**), 9E5R (*env2*-Cbl **42**), 9E5S (*env2*-Cbl **63**), and 9E5T (*env2*-Cbl **64**). Source data are provided with this paper.
